# Natural Deep Eutectic Solvents for Solubility and Selective Fractionation of Bioactive Low Molecular Weight Carbohydrates

**DOI:** 10.3390/foods12234355

**Published:** 2023-12-02

**Authors:** Ignacio Jiménez-Amezcua, Manuel Ignacio López Martínez, Ana Isabel Ruiz Matute, María Luz Sanz

**Affiliations:** 1 Instituto de Química Orgánica General (IQOG-CSIC), Juan de la Cierva 3, 28006 Madrid, Spain; ijimenez@iqog.csic.es (I.J.-A.); mi.lopez@iata.csic.es (M.I.L.M.); ana.ruizl@icsic.es (A.I.R.M.); 2 Pharmactive Biotech Products S.L.U., C/Faraday, 7, 28049 Madrid, Spain

**Keywords:** natural deep eutectic solvents (NADESs), ketoses, aldoses, fractionation, low-molecular-weight carbohydrates

## Abstract

Natural deep eutectic solvents (NADESs) have been shown to be selective and environmentally friendly solvents for the extraction of bioactive compounds. However, studies on the solubility of low-molecular-weight carbohydrates (LMWCs) in NADESs are scarce. In this work, new solubility data of LMWCs in NADESs are provided and a new approach based on the use of these solvents for the efficient fractionation of bioactive carbohydrates was explored for the first time. Several mono- and disaccharides and three NADESs based on choline chloride (ChCl) and different donors (2-ethylene glycol (EtG), glycerol (Gly) and ethanedioic acid dihydrate (Eth)) were considered. While the degradation of carbohydrates, mainly ketoses, was detected with ChCl:Eth due to its acidic nature, ChCl:EtG and ChCl:Gly were found to be useful alternatives for selectively separating bioactive ketoses and their corresponding aldoses (e.g., lactulose/lactose and tagatose/galactose) present in equimolar binary mixtures. In addition, the usefulness of ChCl:EtG for the selective enrichment of lactulose to be used as food ingredient or nutraceutical was proven (from a 25% in the reaction mixture to a 56% in the purified sample). NADESs could be used for the selective fractionation of value-added carbohydrates from interfering sugars for several applications, including food science, engineering or pharmaceuticals.

## 1. Introduction

Low molecular weight carbohydrates (LMWCs) are important components of foods as they belong to the group of basic nutrients involved in nutrition and metabolism. They may be naturally present or may be added to improve the sensory, functional and technological properties of food. In addition, certain carbohydrates present functional properties that are beneficial to human health (i.e., prebiotic effect, glycosidase inhibitors, treatment of conditions related to insulin resistance, etc.). However, in natural sources, these LMWCs coexist with other sugars that may interfere with their bioactive or technological properties [1]. The selective fractionation of these bioactive carbohydrates is essential for their analytical characterization, use in the food industry or the evaluation of their bioactivity [2]. However, this is a challenging process due to the similarity and complexity of their structures as well as their varying concentrations in natural samples.

Several techniques based on the use of membranes (such as ultrafiltration, nanofiltration, etc.) have been proposed for the selective separation of carbohydrates [2]; however, their efficiency for LMWCs is limited. Chromatographic techniques (such as ion exchange chromatography, size-exclusion chromatography, simulated moving bead chromatography, etc.) are highly selective for the fractionation of carbohydrate mixtures with different degrees of polymerization (DP), but the fractionation of LMWCs with the same DP and different monosaccharide compositions or glycosidic linkages remains a challenge [2].

Differences in the solubility of carbohydrates in conventional solvents (ethanol, methanol, 1-propanol, etc.), which cause some of them to precipitate, have been used for their selective separation [3]. Despite the potential utility of these methods, their high solvent consumption and low selectivity have motivated the search for greener and cost-effective alternatives, such as ionic liquids (ILs) or deep eutectic solvents (DESs) [4]. The tunability of these solvents and their unique physicochemical properties (negligible vapor pressure over a wide temperature range, high thermal stability, etc.) open up a range of possibilities for the selective extraction of bioactive compounds such as carbohydrates.

The solubility of LMWCs in ILs has been extensively studied in recent years [5,6,7]. These solvents have also been proposed for the selective fractionation of bioactive carbohydrates such as bioactive ketoses/aldoses [8] and value-added polyols/sugars [1]. However, some studies suggest that, depending on their chemical structure, ILs may be as toxic as or even more dangerous than organic solvents and are more expensive [9].

DESs, a new generation of green solvents, are obtained by the complexation of a hydrogen acceptor (HBA), often a quaternary ammonium salt such as choline chloride, and a hydrogen bond donor (HBD), which can be carboxylic acids, alcohols, amines, polyols, acid amides or carbohydrates [10]. If the compounds that make up the DESs are primary metabolites, such as organic acids, amino acids, choline derivatives or sugars, they are known as natural deep eutectic solvents (NADESs) [11,12]. NADESs have been shown to be environmentally friendly and selective for the extraction of certain compounds, mainly phenolic compounds [13,14,15] and polysaccharides [16,17]. However, to the best of our knowledge, there are limited studies related to the solubility of LMWCs in NADESs (glucose, fructose, sucrose and erythritol) [18,19], while these solvents have not yet been used for the selective fractionation of specific LMWCs. Gomez et al. [20] proposed the combined use of NADESs and microwave-assisted extraction to remove soluble sugars from banana puree to obtain an enriched non-starch polysaccharide fraction.

In the present work, the solubility of various LMWCs (including mono- and disaccharides, aldoses and ketoses) in choline chloride-based NADESs and their usefulness for the separation of bioactive ketoses (i.e., tagatose or lactulose) from their corresponding aldoses (i.e., galactose or lactose) was evaluated for the first time. Finally, the potential of NADESs as a new alternative for the selective separation of carbohydrates in real and more complex samples was considered by applying the proposed method to the enrichment of lactulose in a lactose isomerization reaction mixture.

## 2. Materials and Methods

### 2.1. Standards and Reagents

The following, along with 5-hydroxymethyl-2-furaldehyde (HMF), were purchased from Sigma Chemical Co. (St. Louis, MO, USA). Carbohydrate standards: fructose (Fru), galactose (Gal), glucose (Glc), lactose (La), lactulose (Lu), maltose (Mal), sucrose (Suc), tagatose (Tg) and trehalose (Trh). DES constituents: choline chloride (ChCl), 2-ethylene glycol (EtG), glycerol (Gly) and ethanedioic acid dehydrate (Eth).

### 2.2. Synthesis and Characterization of NADESs

NADESs were synthesized as indicated by Hakkinen and Abbott [18] with slight modifications. In brief, HBD (EtG, Gly and EthAc) were mixed with ChCl at 2:1 molar ratio for ChCl:EtG (NADES1, ethaline) and ChCl:Gly (NADES2, glyceline) and 1:1 molar ratio for Eth:ChCl (NADES3, oxaline) in a water bath at 50 °C under stirring until a homogeneous liquid was formed. Characteristics of these NADESs are shown in Table 1.

Dynamic viscosity (η) of synthesized NADESs was determined following the Poiseuille’s law (Equation (1)) as indicated by Geurink et al. [21]. An Agilent 7100 CE instrument with a diode array detector (DAD) and an Agilent bare fused silica (BFS) capillary of 50 µm id, total length 57.5 cm and with acetone as tracer were used. A hydrodynamic pressure (∆P) of 4 bar was applied on one side of the capillary (length (L) 57.5 cm, inner diameter (d) 50 μm) and homogeneously filled with the BGE solution. The velocity (ʋ_gem_) of the tracer was calculated by measuring the migration time (t_m_) over an effective length (L_eff_) of 8.5 cm:

(1) $$\eta =(\Delta P\;\cdot \;d^{2})/(32\cdot {ʋ}_{\mathrm{gem}}\;L)=(\Delta P\cdot d^{2})/(32\cdot L_{\mathrm{eff}}/t_{m}\cdot L)$$

The water content of the NADESs was measured using a C20 Compact Karl Fischer coulometer (Mettler Toledo; Ohio, OH, USA) with HYDRANAL^®^-Coulomat AG (Sigma Chemical Co., San Luis, CA, USA) as the reagent for the volumetric titration.

Fourier transform infrared spectroscopy (FT-IR) analyses were also carried out to determine the functional groups of the individual components (ChCl, EtG, Gly and Eth) as well as those of the respective NADESs. For this purpose, a Fourier transform infrared spectrophotometer Perkin Elmer Spectrum Two using an attenuated total reflectance device (FTIR-ATR; Perkin Elmer, Waltham, MA, USA) was used. Spectra were recorded between 400 and 4000 cm^−1^ with 10 scans and a resolution of 4 cm^−1^.

### 2.3. Synthesis of Lactulose

Lactulose was obtained via the isomerization of lactose according to the method of Montilla et al. [22] with some modifications. Briefly, 2 mL of a solution of lactose (250 mg mL^−1^) and powdered egg shell (final concentration: 30 mg mL^−1^) were added to 8 mL of Milli-Q water and kept at 125 °C under reflux for 4 h. The reaction was stopped via immersion in an ice bath, the egg shell was removed via filtration through a 0.4 μm paper filter (Merck KGaA, Darmstadt, Germany) and the sample was lyophilized.

### 2.4. Solubility of LMWCs in NADESs

One gram of each NADESs was added to an excess amount of the individual carbohydrates. These mixtures were magnetically stirred at 200 rpm at 25 °C (and at 45 °C for NADES2) for 24 h and left to stand for a further 24 h at the same temperature. Preliminary experiments were carried out to confirm that the stirring time was sufficient to achieve reproducibility and accuracy and to determine the amounts of each carbohydrate required (from 20 mg to 1000 mg). Aliquots were collected from the upper layer and diluted between 1:1000 and 1:40,000 (*v*/*v*) with Milli-Q water. Samples were analyzed via high-performance liquid chromatography coupled to mass spectrometry (HILIC–MS). All solubility assays were made in triplicate.

### 2.5. Solubility of Binary Mixtures of LMWCs in NADESs

One gram each of NADES1 and NADES2 were, respectively, added to 1:1 (*w*/*w*) binary mixtures containing lactose/lactulose and galactose/tagatose. The samples were stirred at 200 rpm for 24 h and allowed to stand for a further 24 h at 25 °C. Aliquots of each mixture were diluted as required with Milli-Q water and analyzed via HILIC–MS.

### 2.6. NADESs Treatment of the Lactose Isomerization Mixture

NADES1 (0.5 g) was mixed with 500 mg of the lyophilized isomerization mixture and subjected to the same treatment as described in Section 2.5 for the dilution of binary mixtures. Aliquots of the supernatant were analyzed by HILIC–MS.

### 2.7. Evaluation of Carbohydrate Degradation

Color development of NADES1 and NADES3 samples was assessed using Spectra Max Plus 384 Microplaque Reader (Molecular Devices, San Jose, CA, USA) through an absorbance at 420 nm, according to the method of Meydav et al. (1977) [23]. Samples were diluted (from 1:1 to 1:10 (*v*/*v*)) in sodium phosphate buffer (PBS, 1 mM; pH, 7.02; control value).

The formation of HMF was determined through high-performance liquid chromatography using an HPLC-UV 1100 series with a G1314B VWD detector (Agilent Technologies, Santa Clara, CA, USA), an XDB-C18 column (Zorbax^®^, 5 µm particle size and 80 Å pore size, 150 mm × 4.6 mm i.d., Agilent Technologies) and a binary gradient consisting of methanol/water as indicated by Viñas et al. [24]. An external standard calibration curve (0.025–0.1 mg mL^−1^) was used for quantitative analysis.

### 2.8. HILIC–MS Analysis

Hydrophilic interaction liquid chromatography coupled to mass spectrometry (HILIC–MS) was used for the analysis of LMWCs using a 1260 Infinity II Prime LC System equipped with a diode array detector coupled to a 6125 single quadrupole mass detector (Agilent Technologies, Santa Clara, CA, USA) provided with an electrospray ionization (ESI) source. These analyses were developed by using an ethylene bridge hybrid with trifunctionally bonded amide phase column (150 mm × 4.6 mm; 3.5 µm particle size; 135 Å pore sizep; BEH X-Bridge, Waters, Hertfordshire, UK). A gradient of acetonitrile/water with an addition of 0.1% of ammonium hydroxide at a flow rate of 0.4 mL min−1 was used as follows: from 0 to 30 min, a linear gradient from 20% to 50% aqueous phase, at these conditions, then ramped to the original composition in 1 min, and finally equilibrated for 8 min. The ESI source operated under positive polarity using the following MS parameters: capillary voltage, 4 kV; temperature, 300 °C; nitrogen drying gas flow, 12 L min^−1^; nebulizer (N2, 99.5% purity) pressure, 276 kPa; and fragmentor voltage, 100 V. Mono-sodiated adducts [M+Na]^+^ were formed for the different carbohydrates and monitored in SIM mode. Data were processed using OpenLAB CDS Software (v.2.19.20, Agilent Technologies). External calibration curves of the different LMWCs (0.0001–0.5 mg mL^−1^) were performed in triplicate.

### 2.9. Statistical Analysis

Statistical assays were carried out using Statistica 7.0 (StatSoft Inc., Tulsa, OK, USA). Analysis of variance (ANOVA, Tuckey test) followed to evaluate significance (*p* < 0.05) of differences among solubility values.

## 3. Results and Discussion

NADES1 (ethaline), NADES2 (glyceline) and NADES3 (oxaline) were chosen in the present work based on results reported by Hakkinen and Abbott [18], who determined the solubility of glucose, sucrose and erythritol in these solvents. In addition to their good efficacy in solubilizing carbohydrates, choline-based NADESs are also valued for their biological activity [25]. Characterization of synthetized NADESs was carried out on the basis of their viscosity and water content, considering the relevance of these properties for their use as solvents. As shown in Table 1, the water content was below 0.3% for NADES1 and NADES2, while NADES3 contained 10% of water.

Regarding viscosity, NADES2 showed the highest values (309.2 cP) while NADES1 was the least viscous (35.6 cP). These values agreed with those described by Hakkinen and Abbot [18], except for NADES3, which showed a markedly lower viscosity (137.4 cP) than that reported (767 cP). However, this value was in good agreement with that described by Skulcová et al. [26]. Viscosity of NADESs is associated to the strong hydrogen bonding interactions produced between HBA and HBD. The high viscosity of NADESs is one of the main disadvantages of these solvents as it can reduce mass transfer during extraction. However, the NADESs used in this work showed adequate viscosity values, which makes them promising solvents for carbohydrate solubility studies.

Moreover, FT-IR analyses were carried out to confirm the formation of the NADESs. As shown in Figures S1–S3 (Supplementary Material), spectra of the pure compounds and their eutectic mixtures were obtained and compared, where conformational changes due to intermolecular interactions between HBA and HBD were observed, according to other authors [27,28,29].

### 3.1. Carbohydrate Degradation

Before measuring the solubility of the different LMWCs in the selected NADESs, their potential degradation in these solvents was evaluated. When some carbohydrates were dissolved in NADES3 at 25 °C, a change in color from transparent to brown was observed. On the contrary, color changes were not observed for carbohydrates dissolved in NADES1 and NADES2 at this temperature.

The change in color could be due to the non-enzymatic degradation of the carbohydrates dissolved in NADES3 [18]. To evaluate this reaction, absorbance at 420 nm was measured (Table 2). Before the measurement, 1:2 and 1:6 (*v*/*v*) dilutions in PBS were mandatory for fructose and tagatose solutions, respectively. Under the same conditions, a control assay was performed with each carbohydrate dissolved in PBS; the absorbance values were in all cases lower than 0.059 units of absorbance (UA). A noticeable increase in the absorbance was mainly found for tagatose, followed by fructose, sucrose and lactulose. Aldose solutions, such as glucose and lactose, also showed a slight increase in the absorbance values but was less than for ketoses. Changes in the color of maltose and galactose solutions were not observed.

The degradation of glucose and cellobiose in oxaline at 25 °C has been previously reported, while erythritol remained stable under the same conditions [18], probably because it does not contain a reducing end. A similar behavior has been observed for some carbohydrates dissolved in acidic ILs; it has been described that fructose dissolved in these solvents is more prone to the formation of dehydration products (e.g., HMF) than glucose [7,30].

In this work, HMF was also determined in carbohydrate solutions that presented higher absorbance values (Table 2). The greatest HMF concentrations (ratios above 6%, *w*/*w*) were found for tagatose and fructose in NADES3, followed by sucrose (4.0% *w*/*w*) and lactulose (2.6% *w*/*w*). On the contrary, the production of HMF via the degradation of the aldoses, glucose and lactose was very low (below 0.05%, *w*/*w*). Wu et al. (2018) indicated that, in comparison with neutral or basic DESs, acidic DESs can contribute to the equilibration of a higher proportion of furanose forms, which have been described as the major forms responsible for converting ketoses such as fructose and tagatose into 5-HMF [31]. Based on these results, acidic NADESs could be used as catalysts for these dehydration reactions, mainly for ketoses and, to a lesser extent, for some aldoses, and likewise, for some imidazolium-based ILs [7,32].

Considering these results and to provide reliable solubility data, NADES3 was discarded for the following studies.

### 3.2. Solubility of Carbohydrates at 25 °C

Table 3 shows the solubility data of the different carbohydrates in NADES1 and NADES2 at 25 °C. In general, the solubility of monosaccharides was higher in NADES2, except for glucose, whereas that of disaccharides was higher in NADES1, except for trehalose.

Regarding monosaccharides, significant differences in the solubility of glucose and fructose were observed in both NADESs. The solubility of fructose was higher than that of glucose in NADES2 (245 vs. 135.7 mg mL^−1^). This ketohexose has also been found to be more soluble than glucose in different imidazolium-based ILs [6,7,33], considering its more stable structure and its lower melting temperature (378.1 K vs. 423.1 K for Fru and Glc, respectively) and enthalpy [6]. However, in this work, the solubility of fructose in NADES1 (191 mg mL^−1^) was significantly lower than that of its corresponding aldose (242 mg mL^−1^). Moreover, great differences were observed between the solubility of glucose and its C-4 epimer (galactose) in both NADESs (Glc/Gal: 7.7 in NADES1 and 3.2 in NADES2); the same behavior was observed for fructose and its C-4 epimer (tagatose), although the differences were smaller (Fru/Tg: 2.6 in NADES1 and 1.6 in NADES2). These results point to these NADESs as suitable solvents for the selective fractionation of these isomeric carbohydrates.

As for the disaccharides, large differences in solubility values were observed for both NADESs. In this sense, lactulose (4-O-β-galactopyranosyl-D-fructofuranose) was the most soluble sugar in NADES1 (666 mg mL^−1^), while the solubility of lactose (4-O-β-galactopyranosyl-D-glucopyranose) in this solvent was the lowest (147 mg mL^−1^; Lu/La: 4.5). Similarly, lactulose (135.1 mg mL^−1^) was more soluble than lactose (26.3 mg mL^−1^; Lu/La: 5.1) in NADES2, although solubility values of both carbohydrates were lower than in NADES1. Hakkinen and Abbot [18] observed a similar behavior between sucrose (α-D-glucopyranosyl-β-D-fructofuranose) and cellobiose in ethaline (the former was fourteen times more soluble than the latter). They attributed these differences to the more rigid structure of cellobiose (disaccharide with two symmetrical glucose units linked with a 1-4 glycosidic linkage) that restricts bond rotation, while sucrose has a less symmetrical structure with more conformational freedom in solution, resulting in a higher solubility. However, maltose, which is also constituted by two symmetrical glucose units linked with 1-4 linkage, showed higher solubility values in both NADESs than sucrose. Similarly, the solubility of trehalose (1-O-α-D-glucopyranosyl-α-D-glucopyranose) was significantly higher than that of sucrose in NADES2, although differences were lower in NADES1. It is likely that the different factors such as their folded structure in the solution or the greater hydrogen bond capacity [18,34], among others, could contribute to the solubility of these disaccharides in the NADESs.

Considering that the HBA (choline chloride) of studied NADESs is the same in both solvents, differences in solubility values observed for the different carbohydrates could be attributed to the HBD (2-ethylene glycol in NADESs 1 and glycerol in NADES2).

### 3.3. Solubility of Carbohydrates at 45 °C in NADES2

The effect of the temperature on carbohydrate solubility in NADESs was investigated. While color changes in carbohydrate solutions at 45 °C in NADES2 were not detected, some solutions in NADES1, mainly of ketoses, became darker. Then, NADES2 was selected for the following studies and results are shown in Table 3. As expected, most carbohydrate solubility values were significantly higher with increasing temperature from 25 °C to 45 °C, as previously observed with other solvents [7]. A moderate increase was found for the solubility of tagatose and galactose, which increased by a factor of 1.4 and 1.6, respectively. However, higher differences were observed in the solubility of fructose, lactose and sucrose, which increased by a factor of 3.3, 3.5 and 4.5, respectively. Intermediate increases in solubility values were found for other carbohydrates.

### 3.4. Fractionation of Bioactive Ketoses from Aldoses in Equimolar Binary Mixtures

Some ketoses such as tagatose and lactulose are used as additives or functional ingredients in food, cosmetic and pharmaceutical formulations due to their texturizing and stabilizing behaviors and to their bioactive properties, such as prebiotic effect [35,36]. These bioactive ketoses are usually synthesized from their corresponding aldoses (galactose and lactose) using different catalysts [22,37], which affect their isomerization yields. The remaining aldoses must be removed from the product, requiring an efficient selective fractionation process. Considering the different solubility of aldoses and ketoses in the studied NADESs, the feasibility of these solvents for the selective fractionation of lactulose and tagatose from their corresponding aldoses (i.e., lactose and galactose) in binary mixtures was evaluated.

According to the solubility data shown in Table 3, NADES2 at 25 °C was chosen for tagatose/galactose fractionation because it provided the highest differences between the individual solubility values of both sugars (Tg/Gal: 3.5), which, in principle, could lead to an effective fractionation. For lactulose/lactose, NADES1 at 25 °C was selected, also taking into account the high solubility of lactulose in this solvent.

**Figure 1:**
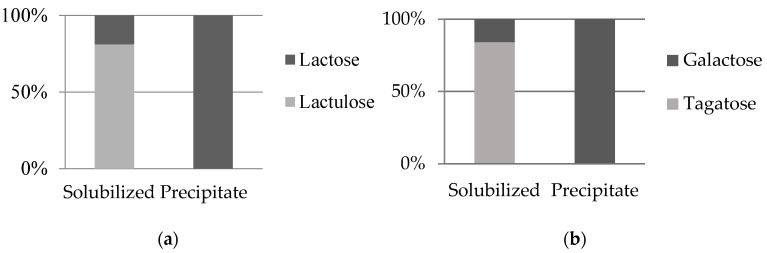
Content (as percentage, %) of lactulose and lactose (**a**) and tagatose and galactose (**b**) in 1:1 (*w*/*w*) binary mixtures in NADES1 and NADES2, respectively, determined as remaining in solution and precipitate after treatment at 25 °C.

Figure 1 shows the solubilized and precipitated percentage of each carbohydrate of the equimolar binary mixtures after NADESs treatment. Regarding the lactulose/lactose mixture (Figure 1a), all lactulose dissolved in NADES1, while only 18.8% of lactose remained in this solvent. Then, lactose could be purely obtained in the precipitate, while lactulose was highly enriched in the solution (77%). Similarly, in the galactose/tagatose binary mixture (Figure 1b), 92.7% of the total tagatose dissolved in NADES2, while only 13.5% of the galactose was found in the solution. High-purity galactose could be separated in the precipitate, while an enriched solution of tagatose (16% galactose; 84% tagatose) was obtained. In both cases, ketoses were more soluble than aldoses, which agreed well with the individual solubility data reported in Table 3. These data point out the feasibility of using these green solvents for ketose/aldose fractionation.

### 3.5. Fractionation of Lactulose from the Isomerization Reaction Mixture

**Figure 2:**
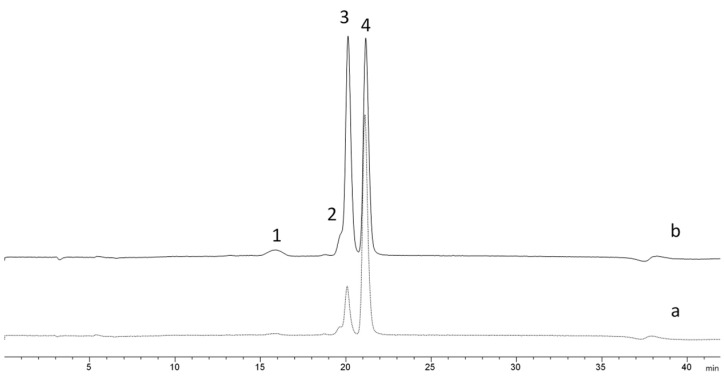
HPLC–MS profile of the isomerization with egg shell of lactose to lactulose before (a) and after (b) treatment with NADES1 at 25 °C. Labeled peaks are as follows: (1) monosaccharides, (2) epilactose, (3) lactulose and (4) lactose.

The efficiency of NADESs as solvent candidates for the fractionation of carbohydrates in real mixtures was evaluated. Lactulose was synthetized via the isomerization of lactose using egg shell as catalyzer; Figure 2a shows the HILIC–MS profile of this reaction mixture. The synthesis yielded 25% of lactulose, which is in good agreement with previously published results [8,22], 14% monosaccharides (glucose and galactose) and 3% epilactose; 60% lactose remained as unreacted sugar in the mixture.

Then, NADES1 at 25 °C, previously chosen as a selective solvent for the lactose/lactulose binary mixture, was used for the fractionation of lactulose from the other isomerization reaction products. As it is shown in Figure 2b, the HILIC–MS profile of carbohydrates in the isomerization mixture after treating with this NADES changed. Lactulose became an abundant carbohydrate in the mixture, while a noticeable reduction in lactose was observed.

**Figure 3:**
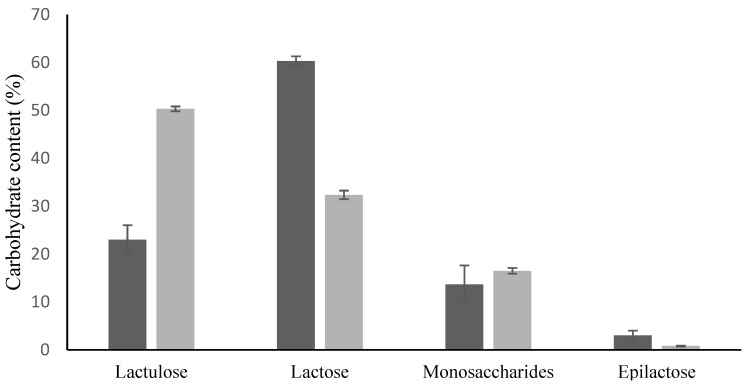
Concentration (%) of lactulose, lactose, epilactose and monosaccharides in the isomerization mixture of lactose to lactulose before (black bars) and after treatment (grey bars) with NADES1.

The extraction yields in NADES1 of the different carbohydrates present in the mixture were determined: while 100% of lactulose was solubilized, only 25% of the available lactose was extracted. A high percentage of monosaccharides (55%) was also dissolved, mainly of glucose, in agreement with the individual solubility of this carbohydrate in NADES1 (Table 3), while 10% of epilactose was also solubilized. Percentages of these carbohydrates before and after treatment are shown in Figure 3. Lactulose percentage showed a 2-fold increase, while lactose and epilactose decreased notably and monosaccharides remained constant after this treatment. Then, the final mixture constituted of 50% lactulose, 32% lactose, 17% monosaccharides and 1% epilactose. These results were quite similar to those achieved by Carrero-Carralero et al. (2015) for lactulose enrichment using ILs (58% lactulose, 31% lactose and 11% monosaccharides), but using a more natural solvent [8].

## 4. Conclusions

In this work, new solubility data of various mono- and disaccharides in selected NADESs (ChCl:EtG and ChCl:Gly) are presented for the first time. Differences were found between the solubility values of carbohydrates as a function of their structure. In general, the solubilities of the ketoses were higher than those of their corresponding aldoses in both solvents. These NADESs proved to be promising alternatives for the selective fractionation of the binary mixtures galactose/tagatose and lactose/lactulose. Furthermore, the results obtained in this work provide the first evidence of the usefulness of ChCl:EtG (NADESs 1) for the enrichment of lactulose in its synthesis mixture. They may contribute to the development of new applications based on the separation of value-added carbohydrates using this new generation of sustainable bio-solvents.

## Figures and Tables

**Table 1:** Viscosity and water content of synthesized NADESs.

	Hydrogen Bond Acceptor (HBA)	Hydrogen Bond Donnor (HBD)	Molar Ratio HBA:HBD	Viscosity (cP)	Water Content (%)
NADES1	Choline chloride (ChCl)	2-Ethylene glycol (EtG)	1:2	35.6 (0.9) *	0.29
NADES2		Glycerol (Gly)	1:2	309.2 (0.9)	0.23
NADES3		Ethanedioic acid dihydrate (Eth)	1:1	143.8 (0.7)	9.96

* Standard deviation in parentheses (*n* = 3).

**Table 2:** Measure of absorbance at 420 nm and 5-hydroxymethylfurfural content (%, *w*/*w*) of carbohydrate/NADES3 mixtures at 25 °C. Absorbance data of carbohydrate solutions in phosphate-buffered saline (PBS) are also included as reference.

LMWCs	Absorbance at 420 nm (A.U.)			HMF (% *w*/*w*)
	Dilution	PBS	NADES3	
Tagatose	1/6	0.0467 (0.0003) *	0.5837 (0.0065)	6.23 (0.68)
Fructose	1/2	0.0491 (0.00017)	0.4631 (0.0018)	6.32 (0.16)
Glucose	1/1	0.0458 (0.0002)	0.1226 (0.0003)	0.02 (0.01)
Sucrose	1/1	0.0477 (0.0002)	0.4752 (0.0002)	4.00 (0.13)
Lactose	1/1	0.0584 (0.0002)	0.0970 (0.0005)	0.040 (0.001)
Lactulose	1/1	0.0587 (0.0001)	0.3595 (0.0004)	2.63 (0.08)

* Standard deviation in parenthesis (*n* = 3).

**Table 3:** Solubility (mg g^−1^) of monosaccharides and disaccharides in NADESs at 25 °C and 45 °C.

	LMWCs	NADES1	NADES2	
		25 °C	25 °C	45 °C
Monosaccharides	Glucose	242 (16) *^,f^	135.7 (0.2) ^c^	364 (1) ^c^
	Galactose	31.4 (0.4) ^a^	43 (4) ^a^	71 (6) ^b^
	Fructose	191 (1) ^d^	245 (5) ^d^	806 (16) ^f^
	Tagatose	72 (2) ^b^	150.0 (0.4) ^c^	214 (7) ^b^
Disaccharides	Lactose	147 (5) ^c^	26.3 (2.9) ^a^	92 (29) ^a,b^
	Maltose	323 (10) ^g^	280 (14) ^e^	630 (90) ^e^
	Trehalose	183 (3) ^d,e^	252 (17) ^d^	503 (13) ^d^
	Sucrose	152 (3) ^c,e^	82 (11) ^b^	368 (15) ^c^
	Lactulose	666 (25) ^h^	135.1 (0.3) ^c^	250 (11) ^b^

* Standard deviation in parenthesis (*n* = 3). ^a–h^ Different letters within the same column indicate significant (*p* < 0.05) differences for the CHs assayed.

## Data Availability

The data used to support the findings of this study can be made available by the corresponding author upon request.

## Supplementary Materials

The following supporting information can be downloaded at: https://www.mdpi.com/article/10.3390/foods12234355/s1, Figure S1. FT-IR spectra of chloride choline (ChCl), ethylene glycol (EtG) and NADES1 (ChCl:EtG), Figure S2. FT-IR spectra of chloride choline (ChCl), glycerol (Gly) and NADES2 (ChCl:Gly), Figure S3. FT-IR spectra of chloride choline (ChCl), oxalic acid dihydrate (Eth) and NADES3 (ChCl:Eth).
