# Supplementary material for: Natural Deep Eutectic Solvents for Solubility and Selective Fractionation of Bioactive Low Molecular Weight Carbohydrates
Source: Foods. 2023 Dec 2;12(23):4355. doi: 10.3390/foods12234355 (PMC10706766; doi:10.3390/foods12234355)

Figure S1. FT-IR spectra of chloride choline (ChCl), ethylene glycol (EtG) and NADES1 (ChCl:EtG).

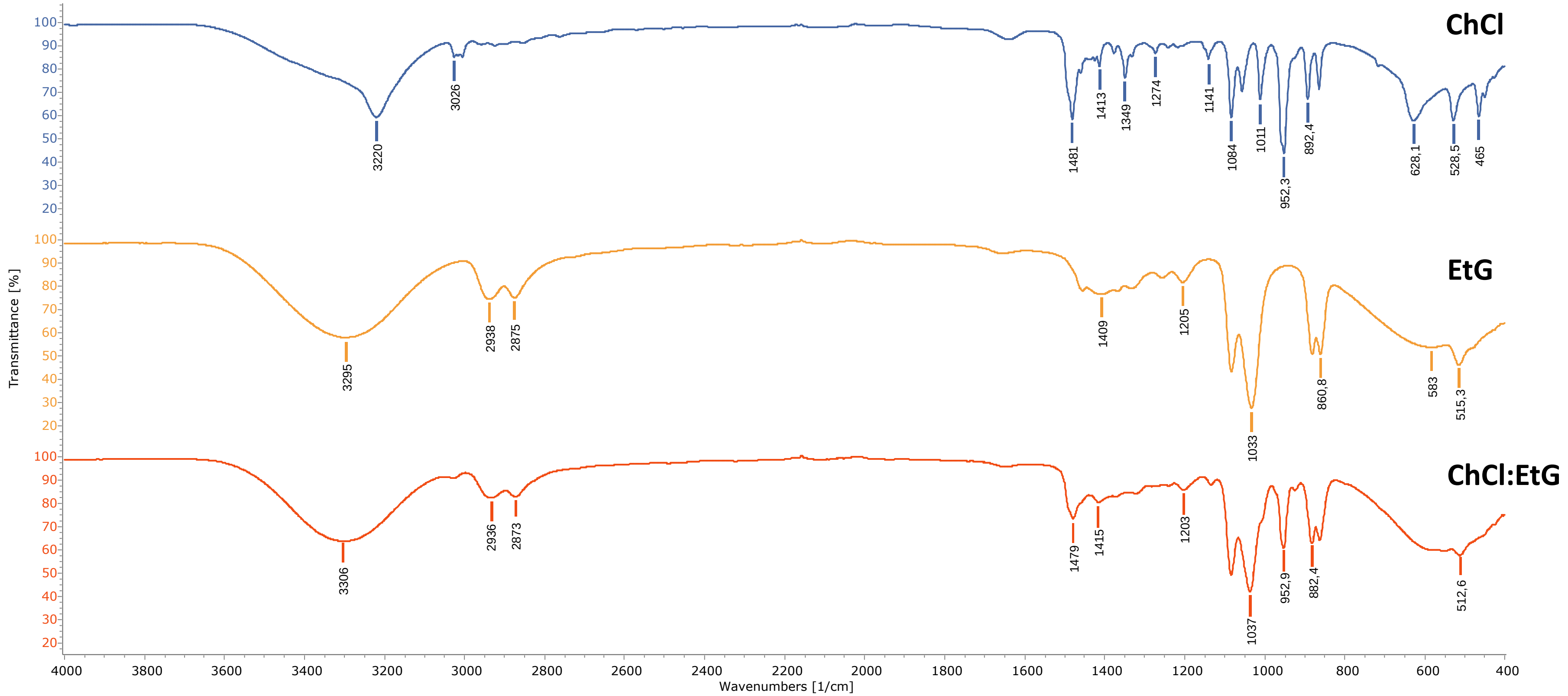

Figure S2. FT-IR spectra of chloride choline (ChCl), glycerol (Gly) and NADES2 (ChCl:Gly).

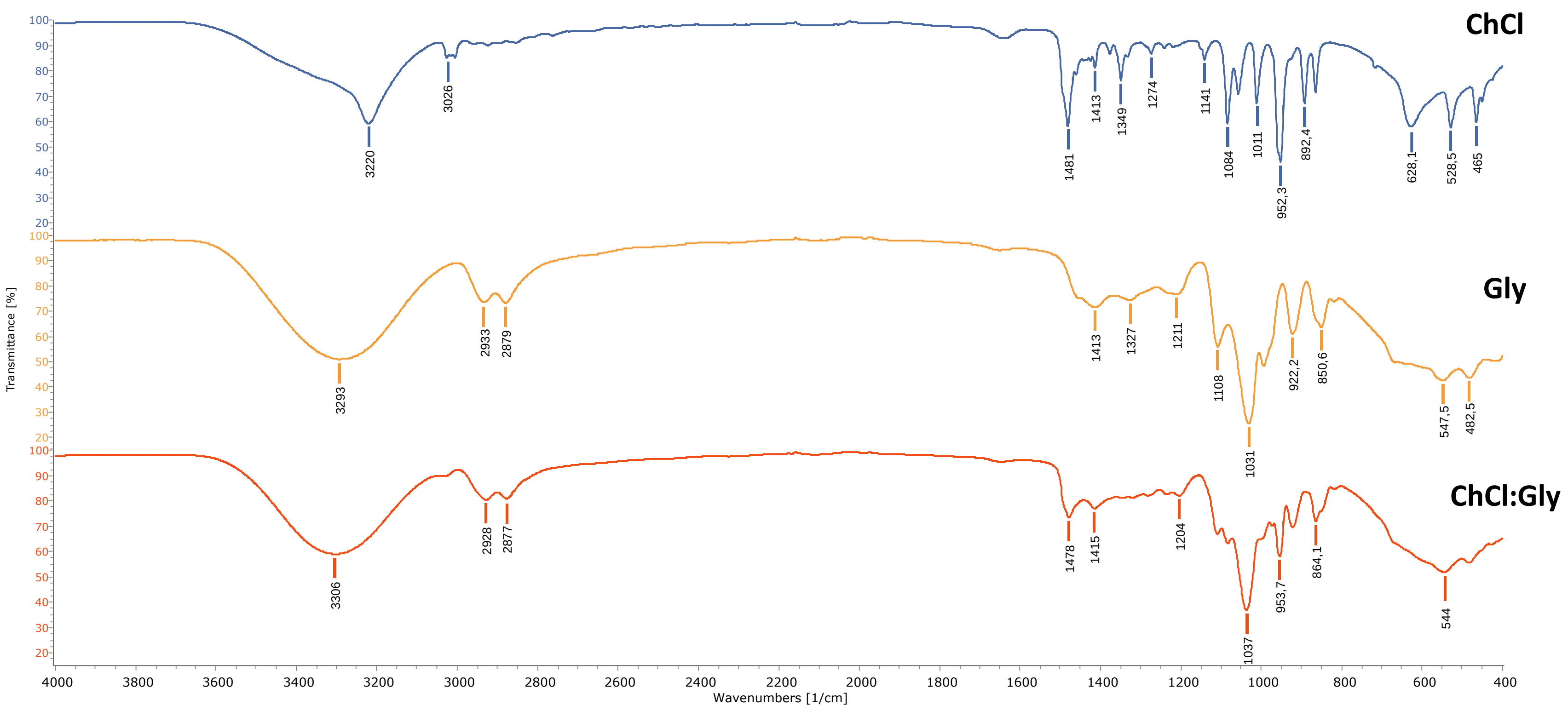

Figure S3. FT-IR spectra of chloride choline (ChCl), oxalic acid dihydrate (Eth) and NADES3 (ChCl:Eth).

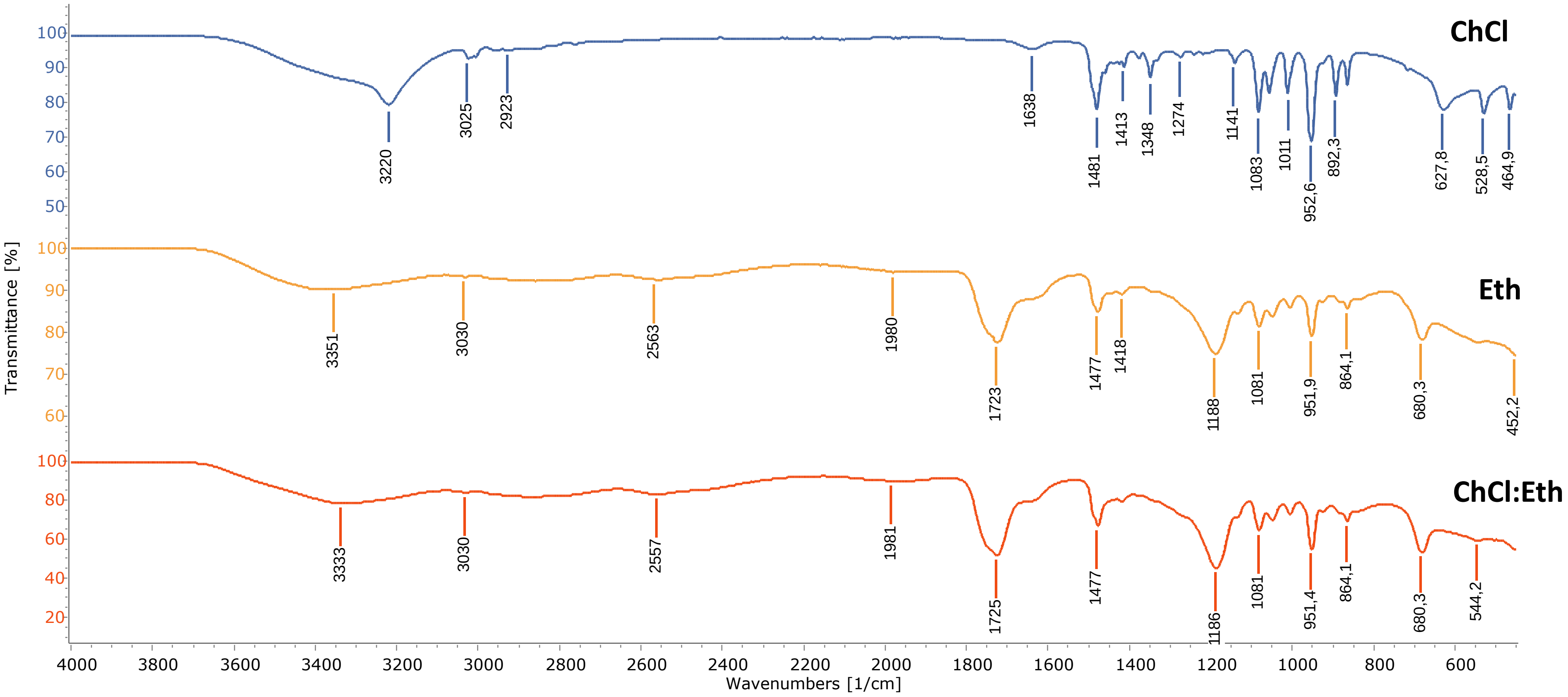

Supplement: Supplementary file 1 [file foods-12-04355-s001.zip › foods-2733903-supplementary.pdf]
